# Chromatin adaptation and histone remodeling as mechanisms of PARP inhibitor resistance and therapeutic vulnerability

**DOI:** 10.3389/fphar.2026.1914271

**Published:** 2026-09-10

**Authors:** Woori Bae, Eun A. Ra, Myon Hee Lee

**Affiliations:** 1 Department of Biochemistry & Molecular Pharmacology, New York University Grossman School of Medicine, New York, NY, United States; 2 Institute for Cell Engineering, Johns Hopkins University School of Medicine, Baltimore, MD, United States; 3 Department of Neurology, Johns Hopkins University School of Medicine, Baltimore, MD, United States; 4 Department of Medicine, Hematology/Oncology Division, Brody School of Medicine at East Carolina University, Greenville, NC, United States

**Keywords:** cancer stem cells (CSCs), chromatin remodeling, histone recycling, INO80-NASP axis, nuclear autoantigenic sperm protein (NASP), PARP inhibitor resistance

## Abstract

Poly (ADP-ribose) polymerase inhibitors (PARPi) have revolutionized the treatment of homologous recombination (HR)-deficient tumors. Nevertheless, their clinical utility is often compromised by the emergence of resistance. Although genetic restoration of HR is a well-established resistance mechanism, emerging evidence suggests that epigenetic and chromatin responses are also central determinants of cell survival under PARPi-induced stress. Indeed, PARP inhibition drives the acute depletion of H3-H4 histones, thereby creating a cellular reliance on efficient histone recycling to ensure genome integrity. The histone chaperone Nuclear Autoantigenic Sperm Protein (NASP) has recently been implicated as a critical regulator in this process by sequestering evicted histones from degradation and promoting chromatin recovery. This NASP-dependent histone turnover may be particularly relevant to cancer stem cell (CSC) biology, as CSCs rely on dynamic chromatin plasticity to switch between phenotypic states and resist therapy. In this review, we focus on the intersection of histone dynamics and CSC survival, with particular emphasis on the ATP-dependent chromatin remodeler inositol-requiring mutant 80 (INO80), the NASP histone maintenance axis, and histone chaperones such as Facilitates Chromatin Transcription (FACT) as potential therapeutic targets. Targeting these chromatin maintenance pathways may provide new opportunities to overcome PARPi resistance and limit the persistence of resistant CSC subpopulations, although further experimental validation is required.

## Graphical Abstract

**Figure:**
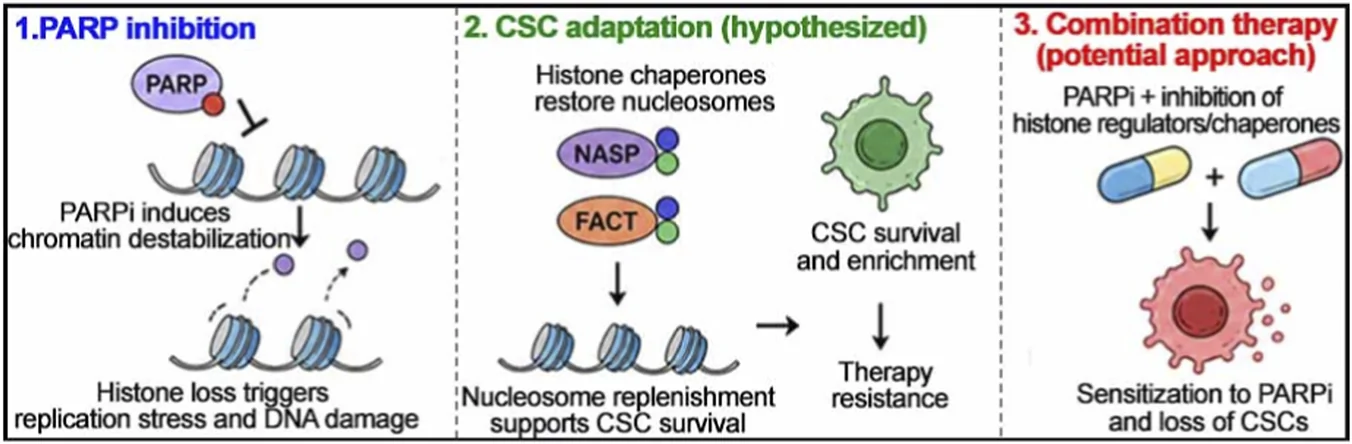

## PARP inhibitors: clinical success and the unsolved problem of resistance

**FIGURE 1:**
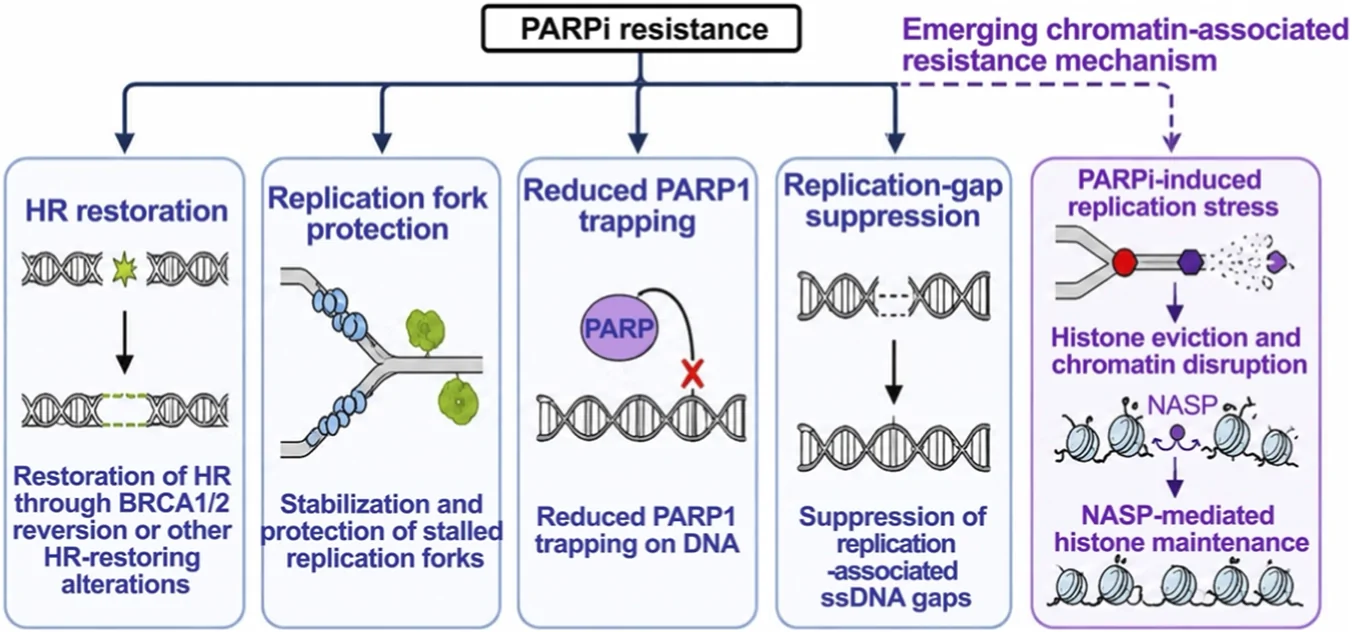
Established and emerging mechanisms of PARP inhibitor resistance. PARPi resistance can arise through multiple well-established mechanisms, including restoration of HR, replication fork protection, reduced PARP1 trapping, and suppression of replication-associated single-stranded DNA (ssDNA) gaps. In parallel, recent evidence has identified chromatin-associated responses to PARP inhibition, in which replication stress is accompanied by histone eviction and chromatin disruption. NASP contributes to histone homeostasis by maintaining the availability of evicted histones and facilitating chromatin recovery. This NASP-mediated histone maintenance pathway is presented as an emerging chromatin-associated mechanism with potential relevance to PARPi resistance. Solid blue connections denote well-established resistance mechanisms, whereas the dashed purple connection highlights the emerging chromatin-associated pathway.

PARPi has revolutionized the treatment of tumors displaying HR deficiency through the principle of synthetic lethality (Kolesnichenko and Scheidereit, 2024; Peng et al., 2025; Lord et al., 2026). However, despite their initial success, both intrinsic and acquired resistance ultimately restrict their long-term therapeutic efficacy (Dias et al., 2021). Traditionally, extensive studies of PARPi resistance have focused heavily on genetic mechanisms, specifically the restoration of HR through second-site mutations in BRCA1/2 or other key repair factors (Norquist et al., 2011; Weigelt et al., 2017; D'Andrea, 2018; Wang et al., 2026). Additional well-established resistance mechanisms include stabilization and protection of stalled replication forks, reduced PARP1 trapping through alterations in PARP1 or associated factors, suppression of replication-associated single-stranded DNA gaps (Andronikou and Rottenberg, 2021; Dias et al., 2021; Cadzow et al., 2024; Lord et al., 2026) (Figure 1). Collectively, these mechanisms preserve genome integrity or reduce replication-associated stress, thereby limiting the cytotoxic effects of PARPi. While these mechanisms are well established, accumulating evidence indicates that they represent only part of a broader adaptive landscape (Cadzow et al., 2024; Seed et al., 2024). Increasing attention is now being directed toward epigenetic and chromatin-based mechanisms that enable cancer cells to tolerate the profound replication stress imposed by PARP trapping (Murai et al., 2012; Andronikou and Rottenberg, 2021; Gopal et al., 2024). In this context, the ability of tumor cells to dynamically regulate chromatin architecture has emerged as an important, yet underexplored, component of the cellular response to PARPi, expanding the landscape of PARPi resistance beyond canonical DNA repair- and replication-associated mechanisms (Figure 1) (Kolesnichenko and Scheidereit, 2024).

## Real-time chromatin dynamics and histone turnover

**FIGURE 2:**
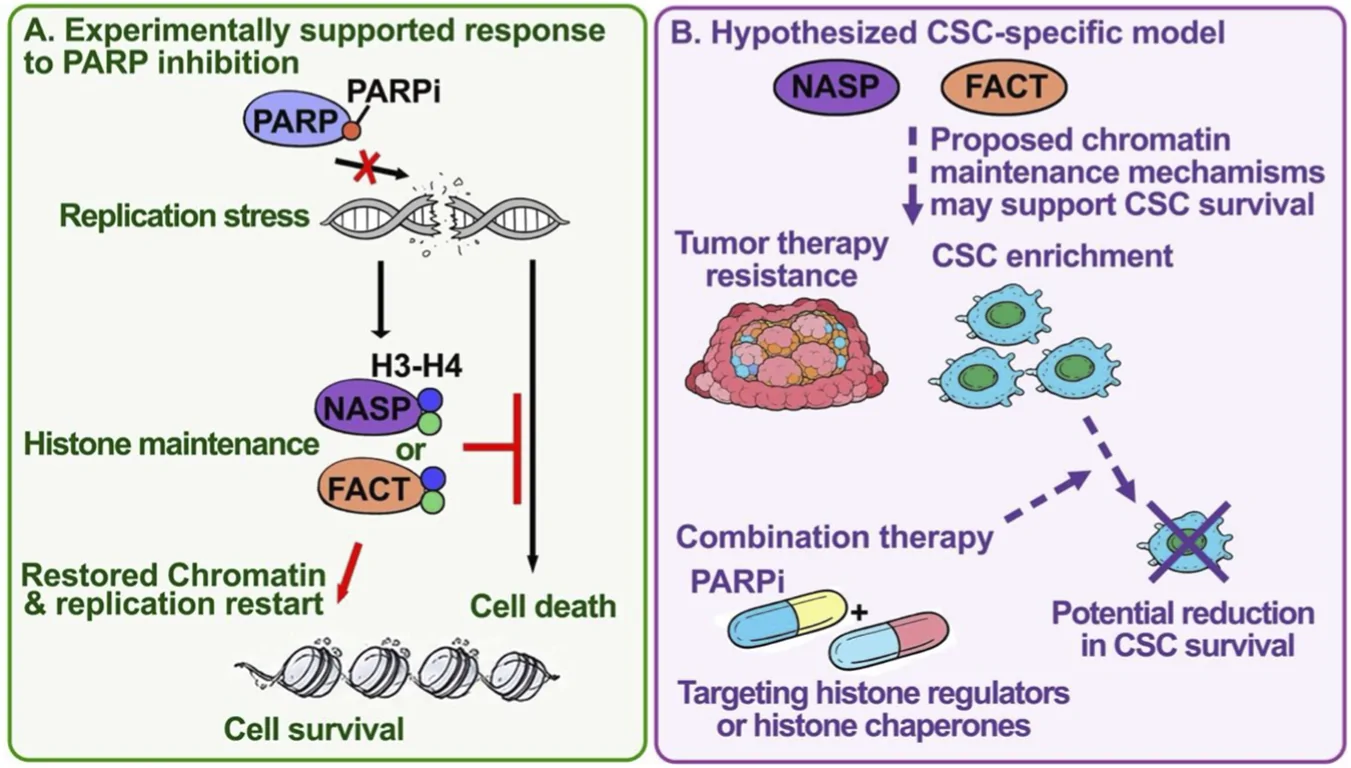
Experimentally supported chromatin responses to PARP inhibition and a hypothesized model linking chromatin maintenance to cancer stem cell survival. **(A)** PARP inhibition induces replication-associated chromatin stress accompanied by H3–H4 histone loss from chromatin. Histone chaperones such as NASP and FACT have established roles in histone homeostasis and chromatin maintenance, respectively, which can support chromatin integrity under replication stress. In contrast, unresolved PARPi-induced replication and chromatin stress can lead to cell death. **(B)** Based on these observations, we propose a CSC-specific model in which chromatin maintenance mechanisms involving NASP and/or FACT may contribute to CSC survival and enrichment during PARPi treatment, potentially facilitating therapy resistance. Accordingly, combining PARP inhibition with agents targeting histone regulators or histone chaperones may reduce CSC survival; however, this proposed therapeutic strategy requires direct experimental validation. Solid arrows in panel A indicate experimentally supported relationships, whereas dashed arrows in panel B denote hypothesized or incompletely validated relationships.

A new paradigm in the field has turned the focus from long-term effects of impaired DNA repair towards real-time epigenetic regulation triggered by PARP inhibition (Langelier and Pascal, 2013; Tubbs and Nussenzweig, 2017; Vaitsiankova et al., 2022). Central to this shift is the understanding that PARPi cytotoxicity is primarily an S-phase event, characterized by the failure to mature nascent DNA strands and the accumulation of single-strand DNA (ssDNA) gaps. The study by Moser et al. (2025) challenged prevailing models by demonstrating that PARP inhibition leads to rapid eviction of histones from nucleosomes, and in particular H3–H4 dimers (Figure 2A). Histone dynamics encompass several distinct but coordinated processes. Histone eviction describes the displacement of histones from nucleosomal DNA, whereas histone recycling refers to the recovery and reuse of evicted parental histones. Subsequent histone deposition restores histones onto DNA, ultimately leading to nucleosome reassembly. Throughout this review, histone turnover is used as the broader term encompassing the dynamic cycle of histone eviction, recycling, deposition, and replacement. This rapid liberation of histones places an immediate burden on genome stability, especially in rapidly proliferating cancer cells in which continuous DNA replication is required (Reveron-Gomez et al., 2018; Hsu et al., 2021; Liu et al., 2023). Therefore, survival under PARPi treatment becomes increasingly reliant on efficient recovery and recycling of evicted parental histones to preserve chromatin integrity. The histone chaperone NASP has emerged as an important mediator of this resistance mechanism. Nuclear Autoantigenic Sperm Protein (NASP) facilitates histone recycling by capturing evicted H3-H4 dimers, maintaining a soluble histone pool, and supporting their subsequent deposition onto DNA during chromatin restoration (Figure 2A). By modulating histone supply, NASP prevents the accumulation of toxic replication-associated DNA damage and allows cancer cells to maintain a plastic chromatin state even in the presence of trapped PARP1 (Xue et al., 2024; Moser et al., 2025).

## PARP1 as a chromatin architect during PARPi response

Emerging evidence indicates that PARP1 does not merely act as a DNA damage sensor but functions as a critical architect of the chromatin landscape. Under physiological conditions, Poly (ADP-ribose) polymerase 1 (PARP1)-mediated PARylation creates a negatively charged environment that promotes the relaxation of compact chromatin, facilitating access of the replication machinery to chromatin (Ray Chaudhuri and Nussenzweig, 2017; Zong et al., 2022). When PARP1 is trapped by PARPi, this PARP-dependent chromatin remodeling is halted. The result is a state of localized chromatin compaction that may physically obstruct replication fork progression (Feng et al., 2019; Andronikou and Rottenberg, 2021; Cong et al., 2021). This biophysical barrier forces the replisome to stall, increasing the frequency of fork reversal or collapse. To overcome this, resistant cells often engage compensatory chromatin maintenance pathways, including NASP-dependent histone recycling, which supports histone homeostasis and chromatin reassembly during replication stress (Frey et al., 2014; Jang et al., 2015; Juhasz et al., 2020; Hewitt et al., 2021; Ramakrishnan et al., 2024; Liu and Xu, 2025; Moser et al., 2025). Chromatin remodelers such as ALC1/CHD1L may also help preserve chromatin accessibility and facilitate DNA damage processing. However, ALC1 should not be described simply as a fork-bypass factor, because its loss sensitizes HR-dependent and BRCA-mutant cells to PARP inhibition (Frey et al., 2014; Jang et al., 2015; Juhasz et al., 2020; Hewitt et al., 2021; Ramakrishnan et al., 2024; Liu and Xu, 2025; Moser et al., 2025). In this context, the resistance is not necessarily about fixing the DNA but about maintaining chromatin accessibility to ensure that replication can proceed despite the replication barriers induced by PARP inhibition. This highlights a therapeutic opportunity. Targeting histone chaperones such as NASP may resensitize resistant tumors by disrupting a critical compensatory mechanism that supports chromatin maintenance during replication stress (Moser et al., 2025).

## NASP-mediated histone homeostasis and resistance

Central to this adaptive response is the histone chaperone NASP, which has emerged as a key regulator mediating histone homeostasis upon PARP inhibition (Moser et al., 2025). NASP employs its highly conserved tetratricopeptide repeat (TPR) domains to recognize a specific peptide motif within the globular core of histone H3. Unlike other histone chaperones that primarily handle newly synthesized histones, NASP shows a strong affinity for evicted H3–H4 dimers, preventing their non-specific aggregation and protecting them from proteasome-mediated degradation (Figure 2A). This ensures that the cell maintains a stable, soluble reservoir of histones that assists in their return to chromatin (Bowman et al., 2016; Bao et al., 2022). The resistance mechanism is supported by the INO80-NASP-PARP1 axis, a coordinated chromatin-remodeling module. Upon PARP inhibition, the INO80 chromatin-remodeling complex is proposed to facilitate histone eviction from regions of PARPi-induced chromatin compaction, thereby promoting chromatin accessibility during replication stress. NASP subsequently captures the evicted H3-H4 dimers, protecting them from proteasomal degradation and maintaining a soluble histone pool for subsequent histone deposition and nucleosome reassembly (Figure 2A). Crucially, loss of NASP causes tumor cells to be hypersensitive to PARP inhibition, further identifying histone supply and recycling as a non-canonical contributor to PARPi resistance (Moser et al., 2025). Unlike BRCA reversion mutations, which restore a repair pathway, NASP-mediated histone maintenance represents a chromatin-based adaptive response that may enable cells to tolerate PARP trapping rather than directly restoring DNA repair. Collectively, these findings suggest that the INO80-NASP chromatin maintenance pathway represents an important chromatin-centered mechanism contributing to PARPi resistance. This pathway appears to operate independently of classical HR reactivation, highlighting a potential therapeutic vulnerability in HR-proficient or PARPi-resistant tumors through the dual inhibition of PARP and histone turnover machinery (Moser et al., 2025).

## Histone chaperones beyond NASP in chromatin adaptation

In addition to NASP, chromatin adaptation during replication stress is coordinated by a broader network of histone chaperones that regulate histone supply, histone deposition, nucleosome assembly, and chromatin restoration. Anti-silencing factor 1 (ASF1) functions as a central histone donor by transferring H3-H4 dimers to Chromatin Assembly Factor-1 (CAF-1) during replication-coupled chromatin assembly or to histone cell cycle regulator, HIRA, during replication-independent H3.3 deposition, thereby preserving chromatin integrity during DNA replication and repair (Reveron-Gomez et al., 2018; Hsu et al., 2021; Liu and Xu, 2025). Facilitates Chromatin Transcription (FACT) further contributes to chromatin maintenance by facilitating histone exchange during transcription and replication while protecting replication fork stability under replication stress (Abe et al., 2011; Prendergast et al., 2020). Although direct evidence linking ASF1, CAF-1, or HIRA specifically to PARPi resistance remains limited compared with NASP (Moser et al., 2025), their established roles in histone recycling and chromatin maintenance suggest that they may also contribute to cellular adaptation following PARPi-induced chromatin destabilization. Defining the functional relationships among these histone chaperones will therefore be important for understanding how chromatin maintenance networks influence therapeutic resistance.

## Chromatin adaptability and histone dynamics in cancer stem cells

Cancer stem cells (CSCs) are a small subpopulation of tumor cells characterized by self-renewal, phenotypic plasticity, and long-term propagation potential (Chu et al., 2024; Lee et al., 2025). A defining hallmark of CSCs is their capacity to reprogram transcriptional and epigenetic states in response to therapeutic stress and microenvironmental pressures (Najafi et al., 2019; Thankamony et al., 2020). Rather than remaining in a fixed differentiated state, this plasticity enables CSCs to reversibly switch among stem-like, proliferative, and invasive phenotypic states, an essential feature of their persistence after standard therapies and their involvement in tumor relapse (Thankamony et al., 2020; Bisht et al., 2022).

At the molecular level, this plasticity is maintained through high rates of histone turnover and nucleosome remodeling (Jin and Felsenfeld, 2007). Histone variants such as H3.3 and H2A.Z are specifically enriched at regulatory regions, including active promoters and enhancers, where they facilitate transcriptionally permissive chromatin and rapid nucleosome turnover (Chen et al., 2014). Functional studies in CSC models have demonstrated that perturbations of these histone variants profoundly impact CSC fate decisions. For instance, in glioblastoma stem-like cells, depletion of H3.3 reinforces an undifferentiated state, while its restoration promotes lineage commitment (Pathania et al., 2017). Similarly, H2A.Z.1 has been shown to regulate cell cycle progression and epithelial–mesenchymal transition (EMT), thereby contributing to tumorigenesis in hepatocellular carcinoma models (Yang et al., 2016).

Together, these results suggest that CSCs may exploit unique histone dynamics to maintain an undifferentiated and therapy-resistant status (Figure 2B). In line with these findings, genome-wide epigenetic studies and recent reviews suggest that CSCs may adopt a relatively less compact chromatin structure compared with their differentiated counterparts (Völker-Albert et al., 2020; Verona et al., 2022; Lee et al., 2025). This more relaxed chromatin structure allows developmental pathways to be activated or silenced in a reversible manner, thereby enhancing CSC adaptability and resilience under stress conditions. As a result, CSCs can rapidly transition between distinct proliferative and phenotypic states in response to environmental cues. Therefore, these findings highlight histone dynamics as an important component of CSC identity, plasticity, and maintenance (Wang et al., 2025).

Recent studies suggest that histone recycling and chromatin remodeling are important adaptive mechanisms that enable proliferating cancer cells to tolerate PARPi-induced chromatin stress (Liu and Xu, 2025; Moser et al., 2025). Likewise, accumulating evidence demonstrates that CSCs rely on dynamic chromatin organization and histone dynamics to maintain stemness and phenotypic plasticity (Thankamony et al., 2020; D'Accardo et al., 2022; Lee et al., 2025; Wang et al., 2025). Together, these observations raise the possibility that histone recycling may also contribute to CSC survival during PARPi treatment. However, direct experimental evidence supporting this possibility is currently lacking. In particular, whether the coordinated INO80–NASP–FACT chromatin maintenance axis is preferentially engaged in CSCs during PARPi treatment has not yet been experimentally demonstrated. Therefore, the proposed link between NASP-mediated histone recycling and CSC persistence should be regarded as a mechanistic hypothesis rather than an established mechanism. Future studies using CSC-specific experimental models will be necessary to determine whether histone recycling represents a critical dependency underlying CSC survival and therapeutic resistance following PARPi treatment.

## Limitations of PARPi and combination strategies

While PARPis have proven to be potent therapeutics in HR-deficient tumors, their impact on CSCs remains multifaceted and context-dependent (Gillespie et al., 2023). In ovarian cancer models, PARPi exposure has been shown to specifically enrich CD133+ and CD117+ subpopulations, closely linked to tumor-initiating capacity alongside the induction of pluripotency-associated transcription factors, such as SOX2 and OCT4 (Bellio et al., 2019; Nwokolo et al., 2025). Furthermore, in triple-negative breast cancer, a minor subpopulation of cancer stem-like cells with elevated levels of RAD51 exhibits intrinsic resistance to PARP inhibition (Liu et al., 2017). This emphasizes a major limitation of PARP-targeted therapy: CSCs may survive or acquire adaptive characteristics during treatment, thereby maintaining the regenerative capacity of the tumor. Consequently, combining PARPis with strategies targeting CSC survival and plasticity may provide an approach to improving the durability of therapeutic responses (Bhamidipati et al., 2023). Targeting the INO80-NASP axis may therefore warrant investigation as a potential strategy for limiting adaptive survival mechanisms in CSCs. While genetic mutations provide permanent resistance, the epigenetic plasticity of CSCs allows for transient drug tolerance (He et al., 2024). Disrupting histone chaperone networks that underpin this plasticity may constrain CSCs into a more vulnerable chromatin state, thereby limiting phenotypic switching and increasing their sensitivity to PARP inhibition. Recent studies demonstrate that PARP1 directly modulates chromatin architecture and transcriptional responses, and that its inhibition triggers profound epigenetic remodeling in surviving tumor cells (Pinton et al., 2022; Lu et al., 2024). These findings further underscore the rational approach of targeting the dual vulnerability of CSCs, which involves compromising both DNA repair fidelity and chromatin integrity.

## Targeting DNA repair fidelity and chromatin integrity

A prime example of this strategy is the targeting of RAD51, a critical mediator of homologous recombination. Suppression of RAD51 has been demonstrated to restore PARPi sensitivity in CSC-enriched breast cancer models, effectively neutralizing their intrinsic repair proficiency (Liu et al., 2017; Cruz et al., 2018; Peng et al., 2025). Beyond DNA repair, metabolic adaptations also represent a key survival axis for CSCs. In ovarian cancer, combination treatment with Astragalus polysaccharides and a PARPi was found to sensitize CSC-enriched populations by blocking mitophagy via the PINK1/Parkin signaling pathway, thereby promoting mitochondrial dysfunction and cell death (Peng et al., 2024). Similarly, combining a DNA-methyltransferase inhibitor (DNMTi) with a PARPi produces synergistic antitumor activity irrespective of BRCA mutation status by enhancing PARP trapping and perturbing DNA repair signaling (Pulliam et al., 2018). Epigenetic modulators, such as BET inhibitors (BETis), also synergize with PARPi by repressing Bromodomain-containing protein 4 (BRD4)-dependent transcriptional programs, thereby inducing a state of functional HR deficiency (Yang et al., 2017; Sun et al., 2018). At a more fundamental level, PARP inhibition itself has also been reported to contribute to cellular chromatin remodeling dynamics (Andronikou and Rottenberg, 2021; Zong et al., 2022; Nosella et al., 2024). Since CSCs rely heavily on epigenetic plasticity to maintain stemness and adapt to stress, they may be particularly vulnerable to therapeutic strategies that simultaneously target PARP signaling and chromatin organization.

The histone chaperone FACT is one of the chromatin regulators of particular interest in CSC biology (Abe et al., 2011; Sankar et al., 2026). FACT repositions H2A–H2B dimers during transcription and has been implicated in gene expression programs related to self-renewal and stem-like identity (Figure 2A) (Prendergast et al., 2020). FACT expression has been reported to be elevated in several cancers, including pancreatic cancer and glioblastoma, where it has been associated with stemness-related programs. Moreover, pharmacological inhibition of FACT using CBL0137 decreases self-renewal capacity, sphere formation, clonogenic potential, and therapeutic resistance (Burkhart et al., 2014; Tallman et al., 2021). Beyond its established function in transcription elongation, FACT is also indispensable for resolving replication stress by preventing helicase disengagement and maintaining replication fork stability (Prendergast et al., 2020). Since PARP inhibition amplifies replication-associated DNA damage, tumor cells may become increasingly reliant on FACT-dependent chromatin maintenance. Accordingly, combining PARP and FACT inhibition represents a potential therapeutic strategy that warrants further investigation, particularly in PARPi-resistant and CSC-enriched tumors. Collectively, these findings suggest that histone dynamics are not merely a background feature of tumor cells but represent an important component of CSC identity, plasticity, and maintenance.

Recent evidence linking PARP inhibition to NASP-mediated histone turnover in bulk cancer cells provides an additional framework for considering how histone homeostasis may influence CSC biology (Moser et al., 2025). Given the established role of NASP in maintaining histone homeostasis during PARPi-induced replication stress, CSCs may exhibit increased dependence on histone supply pathways, including NASP-mediated histone recycling, because of their high demand for chromatin remodeling and nucleosome turnover. However, whether this dependency is selectively enhanced in CSCs remains to be established experimentally. Exploiting this dependency therapeutically may provide a strategy to target PARPi-resistant CSC populations, which have been reported to expand following PARPi treatment (Lee et al., 2025).

## Therapeutic perspectives and future directions

The emerging understanding of chromatin adaptation has expanded current models of PARPi resistance beyond the restoration of homologous recombination. Recent studies indicate that maintenance of chromatin integrity through histone recycling represents an important adaptive response to PARPi-induced replication stress (Liu and Xu, 2025; Moser et al., 2025). Among these mechanisms, NASP-mediated histone recycling has recently emerged as a promising area for further investigation because of its role in maintaining histone homeostasis following PARPi-induced histone eviction (Bowman et al., 2016; Bao et al., 2022; Moser et al., 2025). Nevertheless, whether this pathway represents a universal mechanism of resistance across different tumor types or is preferentially utilized by CSC populations remains to be determined.

Future studies should therefore investigate how NASP interacts with other histone chaperones, including ASF1, CAF-1, HIRA, and FACT, as well as ATP-dependent chromatin remodeling complexes under therapeutic stress (Hsu et al., 2021; Liu and Xu, 2025). Defining whether these pathways cooperate, compensate for one another, or exhibit tissue-specific functions will improve our understanding of chromatin adaptation during PARPi treatment and may reveal new opportunities for rational combination therapies targeting both DNA repair and chromatin maintenance (Andronikou and Rottenberg, 2021; Moser et al., 2025). Such strategies may ultimately provide more durable suppression of PARPi-resistant tumors while limiting the persistence of therapy-resistant CSC populations (Bhamidipati et al., 2023; Lee et al., 2025). One promising therapeutic strategy that merits further investigation is the combination of PARPi with pharmacological inhibition of FACT using CBL0137 (Figure 2B) (Burkhart et al., 2014; Tallman et al., 2021). Given the established role of FACT in replication fork protection and chromatin maintenance, simultaneous inhibition of FACT and PARP may further compromise adaptive chromatin maintenance during PARPi-induced replication stress (Prendergast et al., 2020). Whether this approach promotes replication failure, preferentially eliminates therapy-resistant tumor cells, and improves long-term therapeutic responses remains to be established experimentally. Beyond FACT-targeted approaches, future therapeutic landscapes will likely involve combining PARPi with additional epigenetic modulators, including BETi or DNMTi, for which preclinical studies have reported synergistic activity (Yang et al., 2017; Pulliam et al., 2018; Fehling et al., 2020).

Despite their therapeutic potential, chromatin-directed combination strategies face several challenges that will need to be addressed before clinical translation. Histone chaperones and chromatin remodelers perform fundamental functions in chromatin organization and genome maintenance, raising concerns that their systemic inhibition could also affect normal proliferating cells. Therefore, the therapeutic feasibility of targeting these pathways will depend on establishing sufficient tumor selectivity and defining therapeutic windows that permit disruption of tumor-specific chromatin dependencies without excessive toxicity. The development of biomarkers that identify tumors with increased dependence on histone homeostasis, chromatin remodeling, or replication-stress adaptation will also be important for patient stratification. In addition, the pharmacological tractability, tissue distribution, and tolerability of agents targeting individual chromatin regulators will need to be evaluated in appropriate preclinical models. Together, these considerations underscore the importance of defining context-specific chromatin dependencies and establishing the efficacy, selectivity, and safety of chromatin-directed combinations before their clinical potential can be determined.

These combinatorial strategies are intended to simultaneously perturb multiple processes associated with CSC survival, including DNA repair fidelity, stemness-associated transcriptional programs, and global transcriptional flexibility. Targeting regulatory networks involved in histone availability and chromatin accessibility, including NASP-mediated histone maintenance, may therefore represent one approach to limiting adaptive responses associated with treatment resistance (Figure 2B). Future research should therefore prioritize identifying the most potent biomarkers of chromatin stress and replication vulnerability to better stratify patients who will derive the greatest benefit from these chromatin-directed combination therapies.

Although Moser et al. demonstrated that genetic loss of NASP disrupts histone recycling and compromises the survival of PARPi-resistant tumor cells (Moser et al., 2025), whether this mechanism specifically supports CSC maintenance remains unclear. Future studies should therefore determine whether NASP is preferentially required in CSC-enriched populations and whether its loss impairs self-renewal, stemness, and tumor-initiating capacity independently of its general effects on cell viability. This could be addressed by comparing NASP dependency between CSC and non-CSC populations using sphere-forming assays, limiting-dilution transplantation, lineage-tracing approaches, and single-cell analysis of stemness states following PARPi treatment. In addition, evaluating whether NASP inhibition eliminates residual CSC populations, prevents tumor recurrence, and produces durable PARPi resensitization in patient-derived organoids and *in vivo* resistant tumor models will be important for establishing its therapeutic relevance. Mechanistic studies should further examine whether altered histone recycling, chromatin accessibility, and replication-stress adaptation are selectively enhanced in CSCs and causally linked to their persistence during PARPi treatment.
